# Population Traits and Threats of *Phengaris arion* (Lepidoptera: Lycaenidae) in Western China

**DOI:** 10.3390/insects17101058

**Published:** 2026-10-14

**Authors:** Fengming Lv, Zhaolong Wang, Suqin Shang, Yalin Zhang, Xiushan Li

**Affiliations:** 1 Key Laboratory of Southwest Wildlife Resources Conservation of the Ministry of Education, College of Life Science, China West Normal University, Nanchong 637009, China; lv31213121@163.com; 2 The Station of Qingyang Forest Pest Quarantine and Control, Qingyang 745099, China; wangzhaolong2025@163.com; 3 College of Plants Protection, Gansu Agriculture University, Lanzhou 730070, China; suqinsh@126.com; 4 Key Laboratory of Plant Protection Resources and Pest Management of the Ministry of Education, Entomological Museum, College of Plant Protection, Northwest A&F University, Yangling 712100, China; yalinzh@nwsuaf.edu.cn

**Keywords:** butterfly, conservation, habitat requirements, Mark–Release–Recapture, mowing

## Abstract

The large blue butterfly, *Phengaris arion*, exhibits particular habitat requirements owing to its specialized myrmecophilous association with host ant species, which substantially limits its population size and persistence. To characterize the population characteristics and threatening factors of this species in western China grasslands, we conducted Mark–Release–Recapture (MRR) surveys at a single habitat patch in Qiangtan Village, located in Gansu Province, Jishishan Baoan, Dongxiang and Salar Autonomous County, from 2022 to 2024. We quantified population abundance and structure and assessed anthropogenic and climatic threats to the local population. Our results showed that the local population size of *P. arion* exceeded 1000 individuals (95% CI: 952–1106.07). Adults emerge in July, with flight activity peaking in mid-to-late July. The population exhibited a female-biassed sex ratio of 1.12:1, a maximum adult lifespan of 16 days, and a maximum dispersal distance of 657 m; most individuals remained within an activity radius of 500 m. The studied population is subjected to multiple stressors, including anthropogenic disturbances (e.g., artificial mowing and overgrazing) and extreme climatic events. In particular, hailstorms trigger pronounced declines in adult abundance. Our findings support that implementing targeted mowing restrictions represents a core conservation measure for sustaining local *P. arion* populations.

## Simple Summary

This study investigated the population size, population structure, and threats to *Phengaris arion* within a habitat patch in Qiangtan Village, Jishishan County, Gansu, China. We evaluated threats facing this population and proposed corresponding conservation measures. The results revealed that the local population of *P. arion* exceeded 1000 individuals. Overgrazing and artificial mowing constitute major threats to its survival, and hailstorms markedly reduce adult abundance. Restricting mowing is a key conservation management approach for this butterfly.

## 1. Introduction

Butterflies of the genus *Phengaris* are mostly endangered, as they exhibit an obligate dependence on specific host plants and host ants. Globally, *Phengaris* butterflies are recognized as one of the three flagship butterfly groups, alongside birdswing and monarch butterflies [1]. The genus is widely distributed across the Palaearctic realm, recorded from grassland habitats spanning Europe, Central Asia and East Asia, yet local occurrences are usually highly restricted [2,3,4,5]. Many *Phengaris* populations across Europe have suffered local or even nationwide extinctions [6,7]. *Phengaris* larvae infiltrate ant nests by mimicking ant chemical cues and acoustic signals, inducing worker ants to carry them into the nest. Inside nests, larvae feed on ant brood to acquire nutrients for development, pupate underground, and emerge as adults the following summer [2,8]. By combining chemical deception and acoustic mimicry, larvae imitate signals produced by ant queens and trick host ants into rearing them as royal brood [9,10,11]. *Phengaris arion*, the Large Blue, is threatened across much of Europe and protected under the EU Habitats Directive [12,13,14]. It possesses a highly specialized, obligate life cycle that depends on a specific ant host and a single larval host plant [7]. This species has become a model taxon for exploring the evolutionary origins of butterfly–ant myrmecophily and offers valuable insights into interspecific coevolution within ecological communities [1,15,16,17].

The abandonment of traditional sheep grazing led to the native extinction of *P. arion* in the United Kingdom by 1979; a later reintroduction programme used source populations from Sweden [6,18]. Early conservation attempts failed to establish self-sustaining populations and repeatedly resulted in local extinctions, stemming from poor understanding of its life history, ant associations, and inappropriate habitat management [6]. Severe declines of *Phengaris* butterflies have drawn widespread attention from ecologists, who emphasize that effective conservation requires comprehensive knowledge of species’ biological traits. Mouquet [19] showed that the quantity and spatial configuration of essential resources are key drivers of *Phengaris* population dynamics, particularly for *P. arion* populations occupying habitats capable of supporting high larval survival and adult fecundity. No single conservation strategy works for all species within *Phengaris*, nor for geographically isolated conspecific populations. Habitat management must therefore be tailored to local environmental conditions.

China supports high insect biodiversity and hosts numerous endemic threatened species. Several *Phengaris* species occur in this region, including *Phengaris teleius* (Bergsträsser, 1779), *Phengaris alcon* (Denis & Schiffermüller, 1775), *Phengaris arion* (Linnaeus,1758), and *Phengaris nausithous* (Bergsträsser,1779) [4,5,20]. Existing Chinese studies have applied Mark–Release–Recapture (MRR) to investigate the population biology, distribution, and habitat associations of *P. teleius* [21]. *Phengaris nausithous* is already listed as Class II protected wildlife under China’s National Key Protected Wildlife List. However, information remains limited regarding the life history, distribution, habitat requirements, effects of anthropogenic disturbance, and conservation status of the other native *Phengaris* taxa. To characterize the habitat requirements, population traits, threatening processes, and conservation measures of *P. arion* in China, we carried out a field study for a relatively large local population in Gansu Province, Jishishan Baoan, Dongxiang and Salar Autonomous County (herein Jishishan County). Using MRR and Pollard transect surveys, we systematically examined the population ecology of this species. We identified its core habitat requirements, quantified impacts from anthropogenic disturbance, and diagnosed primary threats. Based on these results, we propose targeted suggestions for its conservation and management strategies.

## 2. Materials and Methods

### 2.1. Study Site

**Figure 1:**
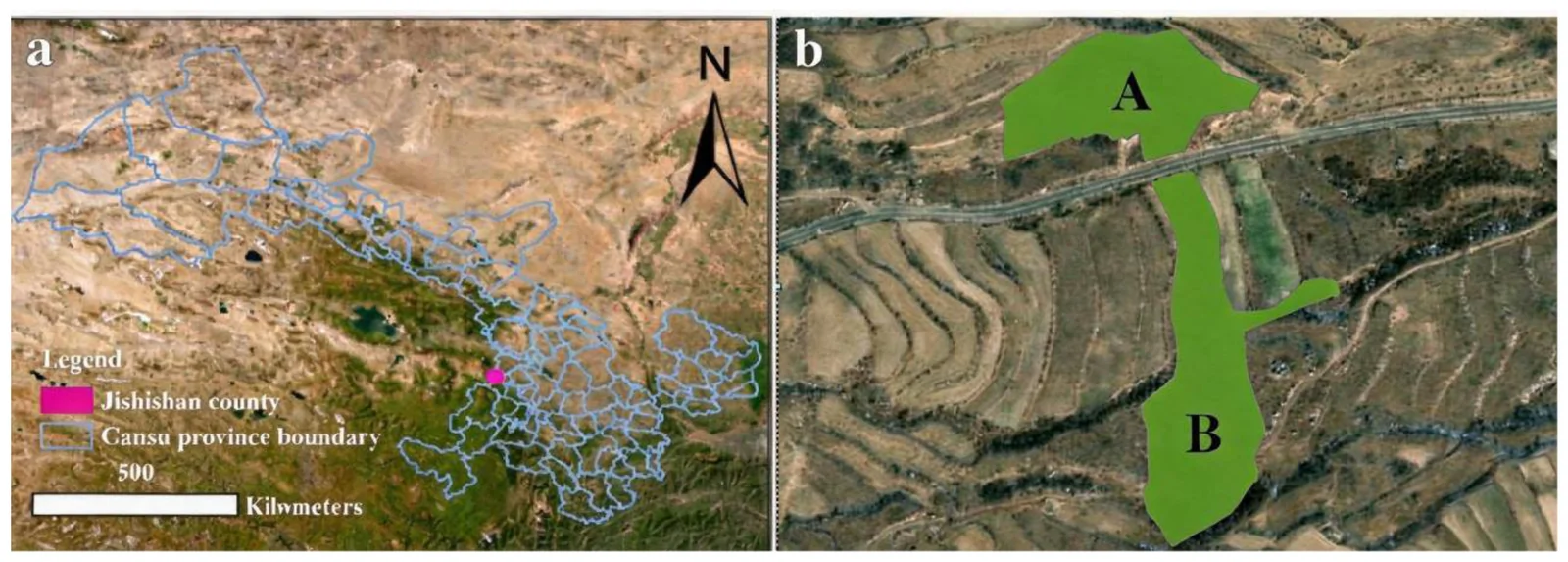
Geographic location of large blue, *Phengaris arion*, survey site in (**a**) Gansu Province, Jishishan Baoan, Dongxiang and Salar Autonomous County with (**b**) specific habitat patches labelled Patch A and B located in Qiangtan Village.

Our study site is located at the intersection of grasslands and farmlands in Qiangtan Village, Jishishan County, Gansu Province. Jishishan County is situated in the southwest of Gansu Province, between 102°41′–103°05′ E and 35°34′–35°52′ N. It is approximately 33 km wide from north to south and 37 km long from east to west, with a total area of 910 km^2^. Qiangtan Village lies on a gentle slope at the eastern edge of Jishi Mountain. Its natural landscape is an ecotone of grasslands and farmlands, with an altitude ranging from 2500 m to 3000 m. The grasslands serve as pastures, while farmland includes the cultivation of rapeseed, wheat, or highland barley. The habitat of *P. arion* surveyed in this study is divided into two patches by an east–west rural road. Patch A is located on the northern side of the road, isolated by a low hill and dominated by farmland and field ridges, while Patch B lies on the southern side and is mainly composed of meadow habitat. The area of both Patch A and Patch B is about 10,000 m^2^ (Figure 1).

**Figure 2:**
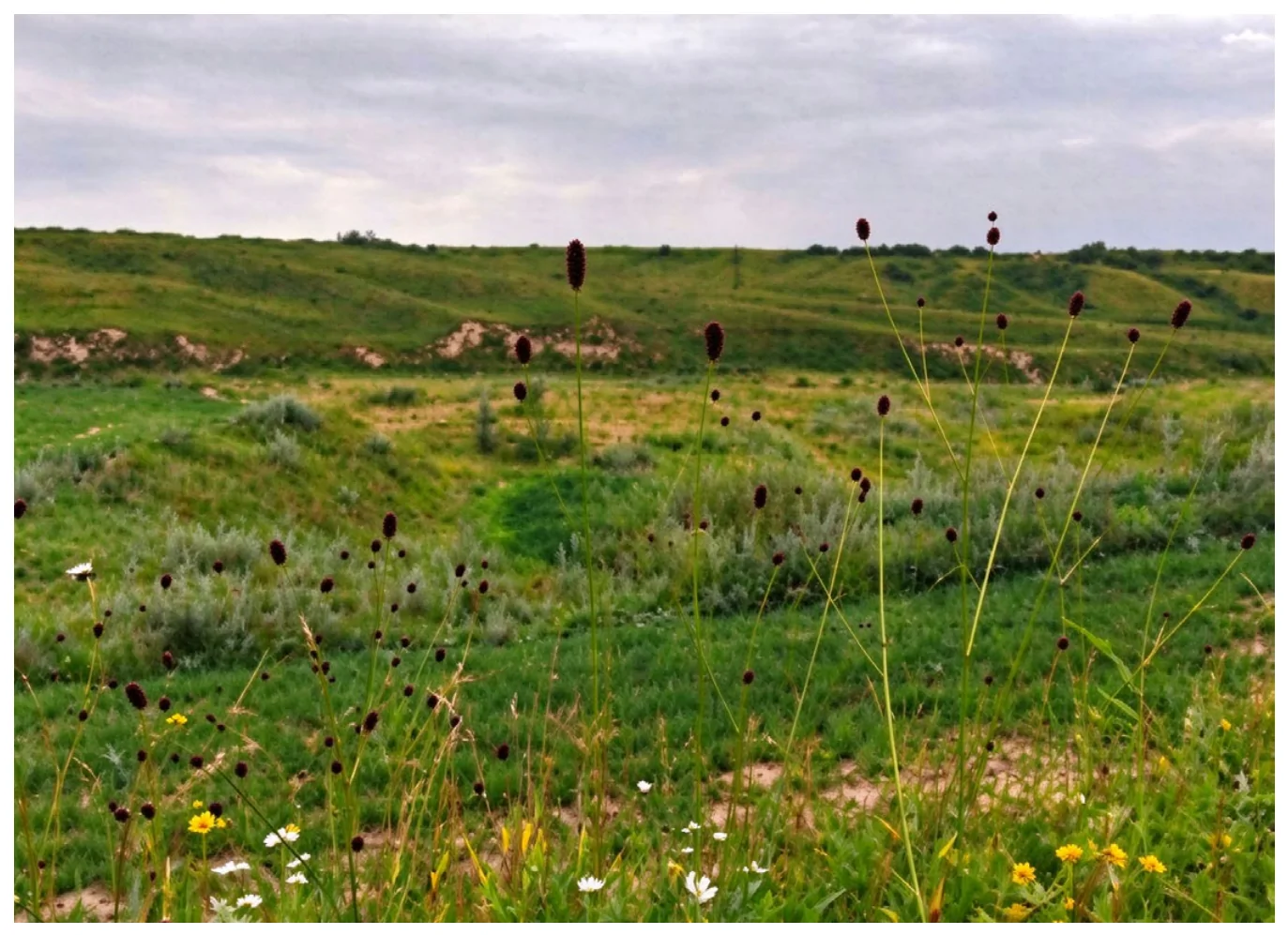
Representative habitat of the study site where the large blue butterfly, *Phengaris arion* (Lycaenidae), and its larval potential host plant, red burnet, *Sanguisorba officinalis* (Rosaceae) where observed.

The habitat of this site is mainly the transitional zone between alpine cold desert meadows and subalpine shrubs. The vegetation coverage ranges from 80% (including farmland and pasture), forming a sparse-tussock type grassland structure (Figure 2). Red burnet, *Sanguisorba officinalis* (Rosaceae), a potential larval host plant of the large blue, occurred in abundance at this field site. Additionally, numerous nectar plant species were observed, such as cranesbill (*Geranium wilfordii*, Geraniaceae), mountain raspberry (*Rubus corchorifolius*, Rosaceae), Japanese thistle (*Cirsium japonicum*, Asteraceae), wild strawberry (*Fragaria vesca*, Rosaceae), Japanese buttercup (*Ranunculus japonicus*, Ranunculaceae), and cow vetch (*Vicia cracca*, Fabacae) (Figure 2). There were nests of red ants present at the site (e.g., *Myrmica lobicornis* and *M. rubis*, Formicidae), providing the necessary conditions for the survival of *Phengaris arion* larvae.

### 2.2. Study Species

**Figure 3:**
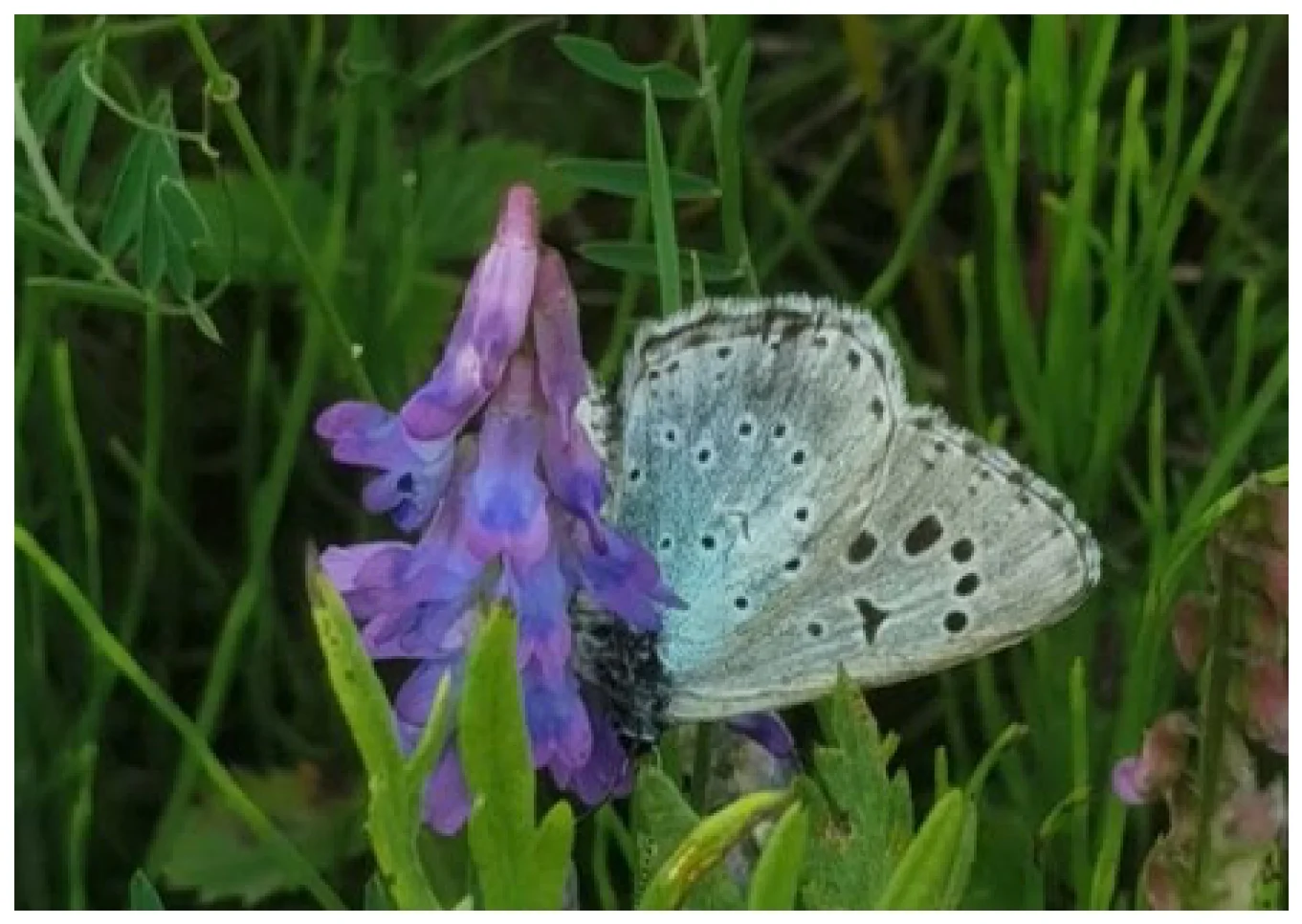
An adult large blue male (*Phengaris arion*) showcasing the ventral wing pattern while nectaring on *Vicia cracca* L. Photos were taken by the authors at Qiangta Village on 15 July 2023.

The upper surface of the male’s wings of *P. arion* is bright indigo blue, with broad black marginal bands, and a row of white spots distributed along the submarginal area (Figure 3). In contrast, the female’s wing colour is relatively dull, presenting a blue–brown hue, and the black marginal bands on the wing edges are wider. There is a series of submarginal double spots (up to 10 and 8 postdiscal spots), but no orange scaling and definitively no tail process [4].

### 2.3. Field Survey Methods

(1) Transect counting

To survey the influence of threat factors, we established one transect with 200 m × 5 m within the core area of *P. arion* in Patch A at Qiangtan Village. Surveys were conducted weekly during the adult flight period. When mowing and hail events occurred, we conducted transect counts before and after these events. We adopted the Pollard walk method for transect counting: walking slowly along each transect at a speed of 2.5 km h^−1^, recording abundance of *P. arion* within 2.5 m on either side of the transect, 5 m vertically above ground.

(2) Sample quadrat survey

Quadrats, 5 m × 5 m in size, were placed in each of the two patches A and B at sites with *S. officinalis*, and the number of flower buds of *S. officinalis* within each quadrat was counted. The average density of host plant *S. officinalis* was 7.8 buds/m^2^ in Patch A and 15.96 buds/m^2^ in Patch B.

(3) Species identification

Species identification of the *P. arion* butterfly used the Monograph of Chinese Butterflies [4], Lycaenidae of China [5], and Butterflies of China [20]. A taxonomy expert on Lycaenidae was invited to identify the species.

To eliminate misidentification between *P. arion* and *P. teleius*, we performed genital dissections of the two butterfly species. Dissection images are provided in Supporting Information S1: Differences between *P. arion* and *P. teleia*.

(4) Behavioural observations

We observed wing expansion following adult eclosion, courtship, and mating behaviours of male and female adults.

Post-emergence flight: Adults emerged from ant nests between 8:00 and 9:00 in the morning, then climbed onto nearby grasses to bask. After their body temperature increased, they expanded their wings and took flight; this process lasted from 30 min to one hour. We recorded the onset and peak timing of adult occurrence throughout the day, as well as flower-visiting behaviour.

Courtship behaviour observations: Two distinct courtship tactics were recorded for this species: patrolling and perching. In the patrolling tactic, males chase females. Once a female accepts the male, the pair alights on grasses or shrubs to mate. In the perching tactic, females perch on grasses or shrubs with wings fully spread, waiting for males to locate them and mount for mating.

(5) Mark–Release–Recapture (MRR)

To estimate population size and other demographic parameters of *P. arion*, we carried out MRR surveys in two habitat patches from 29 June to 6 August 2023. Sampling was conducted on sunny days with light winds (air temperature > 18 °C). Surveys started at 09:00, when adult *P. arion* initiated flight activity, and lasted until 17:00 in the afternoon. Adults were captured with insect nets. Each individual was marked with a numeric ID written on the dorsal surface of the left forewing using a red waterproof marker. Butterflies were handled gently to avoid wing damage and then released immediately at the capture site.

To avoid confusion with the scarce large blue, *Phangaris teleius* (Lycaenidae), we examined diagnostic morphological traits before capture. *P. arion* is larger, with blue wing surfaces and distinct markings. In contrast, *P. teleius* is smaller and dark brown. These two species can be readily distinguished in the field. Our MRR protocol targeted only *P. arion*; no *P. teleius* individuals were captured.

We recorded the Mark ID, sampling date, geographic location, habitat type (grassland, shrubland, farmland), sex (identified by examining terminal genitalia: females possess small circular genital openings, whereas males have claspers), wing condition (fresh/faded colour or slight damage/severe damage), and behaviour (courtship, nectar feeding, flight). For marked individuals recaptured on later days, the same suite of variables was recorded. Individuals recaptured on the same day as marking were released without further recording. For MRR data, see Supporting Information S2.

### 2.4. Data Analysis

All the collected data were processed and analyzed using SPSS 27 and programme MARK. The basic principle for calculating the population size is that the ratio of the number of recaptured marked individuals to the total number of marked individuals is equal to the ratio of the total number of marked individuals to the total population size.

$$N_{t}=\frac{M_{t}+U_{t}}{P_{t}}$$

*U_t_*: the number of newly captured and unmarked individuals at time t (U_t_ = C_t_ − R_t_), Ct: total captured at occasion t, Rt: recaptures at occasion t;
*P_t_*: the capture probability at time t (derived from fitting multiple survey data through the CJS model, and it is not a fixed value);
*M_t_*: denotes the number of marked individuals captured at the t-th sampling occasion.

Data obtained through MRR can be used to calculate the daily individual birth rate and death rate of adults, the total population size during the entire marking period, thereby analyzing the seasonal dynamics of the population.

Apparent survival probability (Φ) and recapture probability (*p*) were estimated using Cormack–Jolly–Seber (CJS) models implemented in programme MARK via RMark (Version 9.0) [22]. The study comprised 35 daily sampling occasions from 29 June to 5 August. Individual capture histories were collapsed into six temporal bins of approximately six days each to ensure model convergence and parameter identifiability; daily recapture rates were insufficient for interval-specific parameter estimation.

We fitted a biologically motivated a priori candidate set: Φ(.)p(.), Φ(t)p(.), Φ(.)p(t), Φ(sex)p(.), Φ(sex)p(sex), Φ(t)p(t), and Φ(sex + t)p(.). Models with sex effects on *p* were limited to avoid overparameterisation given the available recapture data. Symbol (.) indicates the parameter is constant and time-independent. Model selection followed AICc [23]. Goodness-of-fit was assessed using Fletcher’s ĉ from the global model Φ(t)p(t); overdispersion was mild (ĉ = 1.19), so AICc was retained without adjustment. Apparent survival incorporates both mortality and permanent emigration.

Population size analyses performed using the POPAN package in MARK.

## 3. Results

### 3.1. Adult Behaviour of Phengaris arion

Adults of *P. arion* typically emerge between 09:00 and 12:00 at ambient temperatures near 20 °C. Flight activity commences once the temperature exceeds 20 °C. Following eclosion, adults forage for nectar and engage in chasing interactions within grasslands adjacent to farmlands and rarely cross farmland habitats. Both sexes exhibit relatively limited dispersal capacity and are readily captured. Furthermore, adult behaviour appeared unchanged following capture, marking, and release. Flight activity of this species is constrained by the spatial distribution of *S. officinalis* and other nectar resources, including *Cirsium setosum*, *Medicago sativa*, and *Carduus nutans*.

Our observations documented two distinct courtship tactics: perching and patrolling. After eclosion, newly emerged females feed on nearby flowers, mate, and subsequently locate host plants for oviposition.

Mark–Release–Recapture data revealed protandry: males emerged approximately seven days earlier than females. The maximum recorded dispersal distance was 657 m.

### 3.2. Population Traits of Phengaris arion

(1) MRR data

A total of 516 individuals (243 males, 273 females) were marked, of which 155 (30.0%) were recaptured at least once.

The model fit was acceptable (Fletcher’s ĉ = 1.19). Models incorporating time-varying survival received 99.8% of cumulative AICc weight; Φ(sex + t)p(.) was best supported (weight = 0.66), followed by Φ(t)p(.) (ΔAICc = 1.4, weight = 0.33). Models assuming constant survival received negligible support (ΔAICc > 32). Although Φ(sex + t)p(.) was selected as the best model, the effect of sex on survival probability was weak. We therefore used the simpler model Φ(t)p(.) in the subsequent paragraph to facilitate interpretation, focusing our analysis on the temporal variation in survival probability.

Interval-specific apparent survival estimates correspond to survival between successive temporal bins and varied markedly. Survival peaked during interval 2 (early July; Φ = 0.87) and was lowest during interval 5 (late July; Φ = 0.17). Wide confidence intervals reflect low recapture rates and declining sample sizes across intervals, particularly toward the end of the sampling period. The best model suggested marginally higher female survival, though this effect was weak. Recapture probability was constant (*p* = 0.36, 95% CI: 0.25–0.48) (Table 1).

(2) Population Size

Analyses of the POPAN package yielded an estimated population size of 1026 individuals (95% CI: 952.09–1106.07).

(3) Capture dynamics of *P. arion*

**Figure 4:**
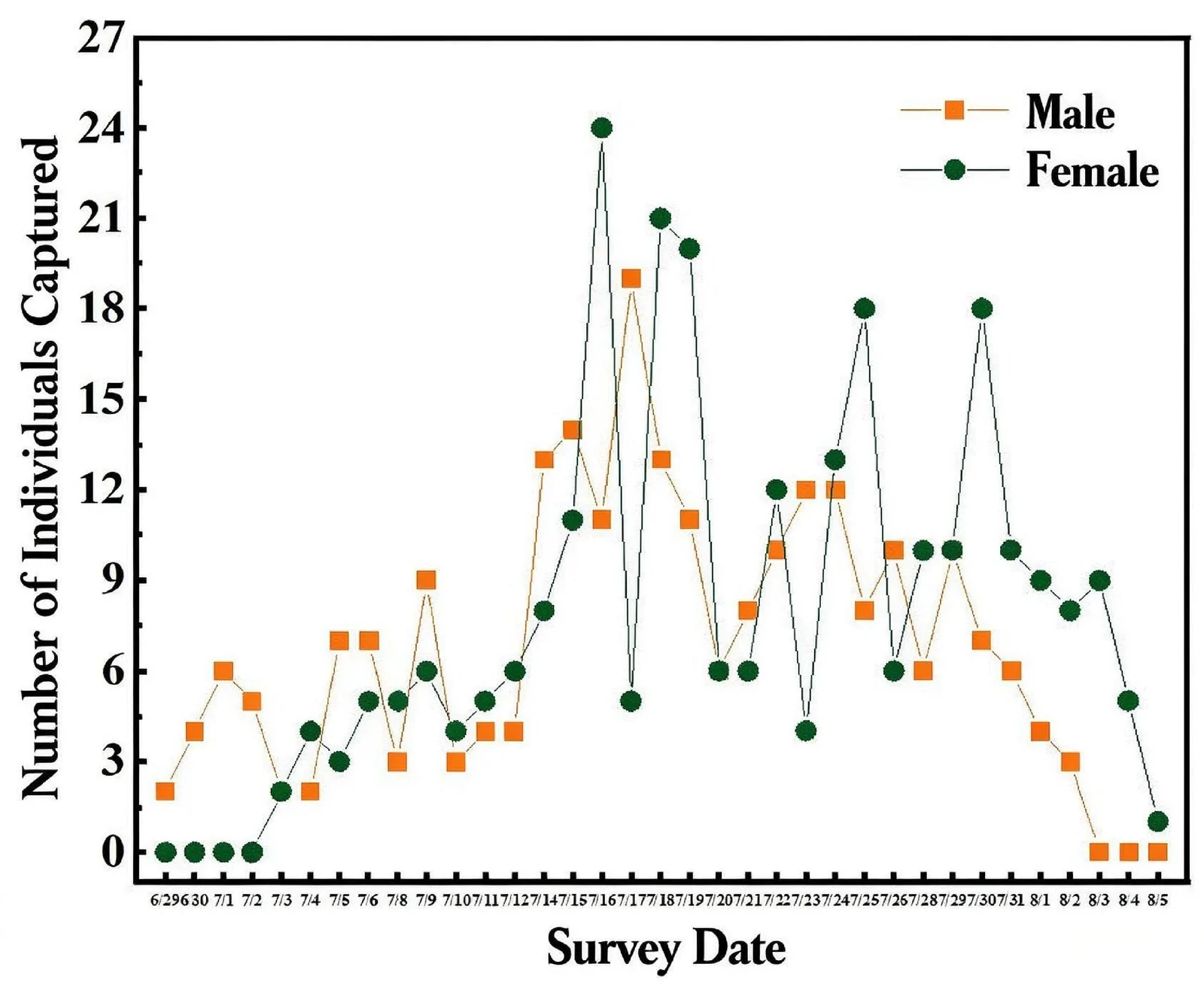
Number of large blue (*Phengaris arion*) individuals captured per sampling day by sex.

Clear sex-specific patterns in adult emergence and population fluctuation were observed (Figure 4). Males of *P. arion* emerged significantly earlier than females, with an approximate one-week time lag. Notably, a pronounced peak of 24 captured females was recorded on 16 July; female numbers remained stable on all other survey dates.

(4) Mobility of *P. arion*

**Figure 5:**
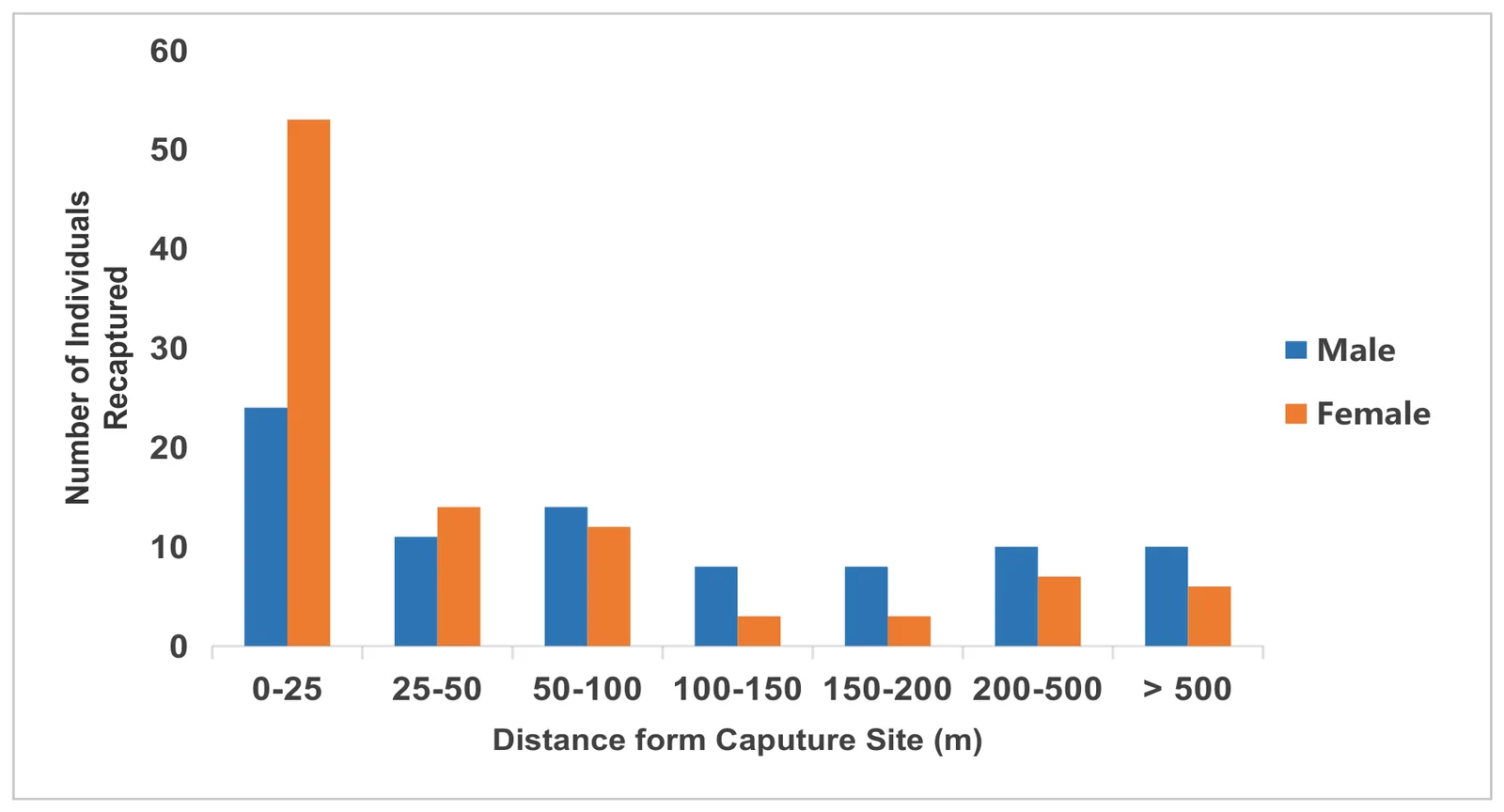
The number of recaptures as a function of distance in metres from the original capture location. Males in this survey have a slightly farther dispersal distance than females, on average.

A <50 m interval represented 53.9% of all marked individuals. The 50–100 m, 100–200 m, 200–500 m, and >500 m intervals accounted for 13.5%, 10.6%, 8.8%, and 12.3% of the total, respectively. Cumulatively, 87.7% of individuals were recorded within a 500 m radius from their marking site. The maximum dispersal distance documented was 657 m. Furthermore, although daily capture counts were higher for females, males achieved longer dispersal distances on average (Figure 5).

MRR data show that *P. arion* moves between Patch A and Patch B, yet inter-patch dispersal is extremely low; only two individuals were recorded crossing between patches, yielding an exchange rate of merely 0.3%. Mowing strongly influences such dispersal behaviour. When host plants and associated nectar flowers within butterfly aggregation sites are cut, adults disperse towards remaining patches with intact *S. officinalis*.

(5) Sex ratio

In total, 516 individuals of *P. arion* were marked: 273 females and 243 males, corresponding to a female-to-male sex ratio of 1.12:1.

(6) Lifespan

MRR data revealed that male adult longevity ranged from 6 to 9 days, with a maximum recorded lifespan of 15 days. For females, average longevity was 6–7 days, and the maximum observed lifespan reached 16 days.

### 3.3. Impact of Mowing and Hail Damage on Population of Phengaris arion

Surveys along a 200 m transect within the core area of *P. arion* showed that mowing and hail events significantly affected butterfly abundance. See Table 2 and Table 3.

After mowing, the number of individuals along the transect declined by 75% on average, which exerted a pronounced impact on its survival.

Data presented in Table 3 show that both the numbers of newly marked and recaptured individuals declined significantly before versus after hail events, demonstrating that hail disturbance substantially reduces adult survival.

## 4. Discussion

### 4.1. Survival Status of the Large Blue Butterfly, Phengaris arion

The population of *P. arion* in Qiangtan Village, Jishishan County is isolated, surrounded by pastures, farmlands and villages, and its survival was impacted by agricultural and pastoral activities. Overgrazing in the extensive surrounding pastures restricts the growth of host and nectar plants, resulting in their low abundance. *S. officinalis* and other plants growing along field margins are subject to irregular mowing between July and August each year. In addition, this site lies at a high elevation (2500 m), at the ecotone between mountains and plains. Hail events frequently occur in summer and damage adult butterflies of *P. alcon*. According to our MRR results, the population size of *P. arion* in Qiangtan Village is approximately 1000 individuals. Although this is not an extremely small population, studies by Hanski have shown that butterfly populations with around 700 individuals may go extinct within two years [24]. Therefore, the *P. arion* population in Qiangtan Village is still at risk of decline, and conservation management is needed for its continued success.

### 4.2. Threats to the Population of the Large Blue Butterfly, Phengaris arion

Like all butterflies, the large blue has many threats at our site. First, rapid economic development has altered land-use patterns: natural grasslands and shrublands have been converted into built-up urban areas, resulting in widespread habitat loss. Since 2016, we have established 50 transects (each 200 m long) for pollinator monitoring across grasslands, forest edges, and farmland–grassland ecotones in Linxia and Gannan Prefectures, Gansu Province. Ten of these transects were later destroyed (the loss rate was as high as 20%) by road construction and tourism development, requiring replacement surveys at alternative sites. Furthermore, local summer weather is a common threat. Hailstorms occur frequently at this time of year and can kill adult butterflies directly. We recorded two hail events during the adult flight peak, which reduced population abundance by an average of 65%.

Yet, overgrazing is the most pressing concern in large blue habitats. Overgrazing is prevalent across grasslands in China, where an estimated 70% of grasslands suffer from overgrazing, and 30% are severely degraded [25]. Our monitoring indicates that *S. officinalis* is nearly absent from heavily grazed grasslands close to towns and villages. In contrast, high host–plant densities occur within lightly grazed ecotones and fenced enclosures. These enclosures are enclosed by wire fences; livestock are excluded in summer, and the tall grass is reserved for winter fodder. Qiangtan Village, our key study site, provides a representative example. Its two habitat patches lie within farmland–grassland ecotones with moderate grazing restriction, supporting high densities of *S. officinalis*, whereas host plant and nectar plants are scarce in the adjacent large, freely grazed pastures. Overgrazing is likely an important driver of its narrow distribution and small population sizes. Thus, agriculture-related mowing constitutes an additional threat at our study sites. Mowing frequently occurs from July to August, which coincides with the oviposition period of adult butterflies and larval development. Repeated mowing decreases host and nectar plant availability, leaving females with few suitable oviposition substrates and both sexes with fewer energy resources. Eggs and early-instar larvae laid before mowing also die as trimmed host plant tissues desiccate after cutting. Finally, it is important to note that *P. arion* is an extremely specialized taxon: it depends not only on a limited number of larval host plant species but also on limited host ant species and abundance, to tend their larvae through development. Overgrazing and mowing may disrupt the butterfly–ant relationship as well.

### 4.3. Population Mobility of the Large Blue Butterfly, Phengaris arion

Butterfly flight capacity generally scales positively with body size: larger individuals tend to travel longer distances [26,27]. Our MRR data indicated that male *P. arion* possessed weak mobility, with a maximum dispersal distance of 635 m, compared with 563 m for females. A previous MRR study on *P. teleius* conducted in Taibai County, Qinling Mountains, recorded a maximum male dispersal distance of 803 and female 632 m [21]. Although *P. arion* has a larger body size than *P. teleius*, its observed maximum male flight distance (635 m) was shorter. This discrepancy may be linked to elevated and ambient temperature. As ectotherms, butterfly flight performance is constrained by thermal conditions. Taibai County lies at ~1500 m a.s.l., whereas Qiangtan Village is located at 2500 m a.s.l., where lower mean temperatures likely restrict flight activity.

Based on MRR records from the two habitat patches, only two individuals were detected moving between patches. The maximum dispersal distance of adults was 635 m. This suggests strong impacts of habitat isolation on this subspecies; adults show high site fidelity and tend to remain within their natal patch. Butterflies confined to highly localized, isolated habitat patches may evolve reduced dispersal capacity. Individuals that disperse away from the patch are lost from the local gene pool, which imposes natural selection favouring genotypes that remain resident [28,29].

Our MRR results revealed that 87.7% of captured *P. arion* individuals were recorded within a 500 m radius of their marking location. Drawing on the home-range concept from animal population ecology [30], we defined the area of a circle with a 500 m diameter as the reference home-range size for this subspecies. In other words, habitat patches larger than 500 m in circular area may be sufficient to support 87.7% of the local population [31].

### 4.4. Limitations Regarding Host Plant and Ant Associations

In Europe, a common host plant of *P. alrion* is *Thymus* spp. (Lamiaceae) [12,32], yet we did not observe any *Thymus* spp. at our Qiangtan Village site. However, we frequently observed *P. arion* adults visiting and ovipositing on red burnet, *Sanguisorba officinalis* (Rosacease), although we located no eggs. Therefore, further observations are required to confirm its host plant at this locality.

We also excavated more than 100 ant nests to identify ant species potentially associated with *P. arion* at our site. Two red ant species were found: *Myrmica lobicornis* Nylander, 1846 and *Myrmica rubra* (Linnaeus, 1758) [32,33,34]. These are known potential host ants for *P. arion*. However, no larvae or pupae of *P. arion* were detected inside any of these surveyed nests.

### 4.5. Conservation Recommendations for the Large Blue Butterfly, Phengaris arion

(1) Mowing restriction

Mowing from mid-July to mid-August coincides exactly with the oviposition period of this butterfly and causes massive mortality of eggs and larvae on host plants. Management guidelines for *Phengaris* butterfly reserves in Romania explicitly recommend postponing mowing until after 25 August to avoid the reproductive season of these butterflies [35]. A MRR study on *P. teleius* further demonstrated that mowing increased butterfly emigration probability from mown grasslands to 22%, compared with only 9% in unmown grasslands. Mowing also reduced mean dispersal distance (mown: 102 m; unmown: 246 m) and resulted in marked population declines [36].

(2) Establish a standardized butterfly monitoring system for long-term tracking of population dynamics

A unified and standardized monitoring approach is important to continue successful conservation management of this and related butterfly species. To those efforts, we have set up 50 monitoring transects for butterflies and wild bees across Linxia Hui Autonomous Prefecture, Gannan Tibetan Autonomous Prefecture and Ruoergai County. Surveys have been conducted intermittently over 10 years, with sampling carried out 5 years (every year four times transect counting) during this period. This monitoring programme should be maintained on a long-term.

## Figures and Tables

**Table 1:** Interval-specific apparent survival probability (Φ) from model Φ(t)p(.).

Interval	Dates	Φ	SE	95% CI
1	29 June–4 July	0.499	0.155	0.228–0.770
2	5–10 July	0.865	0.164	0.289–0.990
3	11–16 July	0.274	0.050	0.187–0.381
4	17–22 July	0.699	0.120	0.412–0.854
5	23–28 July	0.173	0.049	0.097–0.289

Note: Survival for interval 6 (29 July–5 August) is not estimable in CJS models. Because no subsequent recapture occasion exists.

**Table 2:** Comparison of adult *P. arion* counts along transects before and after mowing, 2023.

Date	Before Mowing	Date	After Mowing	Decline%
5 July	15	06 July	5	66.7
12 July	7	14 July	3	57.1
19 July	8	22 July	0	100
26 July	6	28 July	1	83.3
total	36		9	75

**Table 3:** Comparison of *P. arion* abundance along transects before and after hail events.

Date	Period	Marked Individual	Decline%	Recaptured Individual	Decline%
19 July	Before hail	28		35	
20 July	After hail	8		14	
25 July	Before hail	12		14	
26 July	After hail	6		4	
Total		Before hail 40/after hail 14	65	Before hail49/after hail 18	63.3

## Data Availability

The data presented in this study are available upon request from the corresponding author.

## Supplementary Materials

The following supporting information can be downloaded at: https://www.mdpi.com/article/10.3390/insects17101058/s1, Supporting Information S1: Differences between *Phengaris arion* and *teleia*; Supporting Information S2: Mark–Release–Recapture (MMR) data.
