# Supplementary material for: Population Traits and Threats of Phengaris arion (Lepidoptera: Lycaenidae) in Western China
Source: Insects. 2026 Oct 14;17(10):1058. doi: 10.3390/insects17101058 (PMC13705490; doi:10.3390/insects17101058)
Supplement: Supplementary file 1 [file insects-17-01058-s001.zip › Supplementary Information S1_difference between Maculinea arion and teleia(1).pdf]

Here is the difference between *Maculinea arion* and *Maculinea teleia*:

1. *Maculinea arion*

The body is small to medium-sized, with wings that are brownish on the surface and exhibit a purplish-blue iridescence in the medial area. The margins of both the forewings and hindwings are relatively narrow. On the underside, there are two spots in the anterior medial cell region, and the basal area of the hindwing features a flashy blue zone. Wing patterns vary depending on region and temperature conditions.

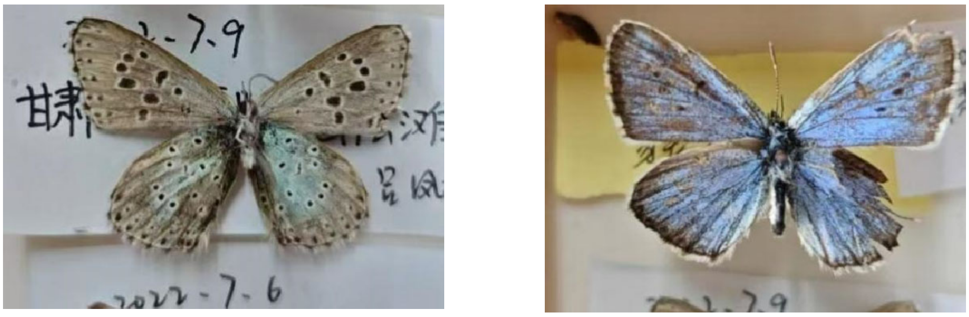

A B

Figure S1. Morphological image of *Maculinea arion*

A. (frontal view); B. (dorsal view);

The Tegumen of the male external genitalia is large and lacks a Saccus. The Valva of the clasping organ is smooth, with a rounded base at the central gap, and the projection above it is straight and slightly upturned. In lateral view, the Valva is more robust than that of *M. teleia*, and the base of the Tegumen is broader. The Phallus is smaller than that of *M. teleia*. The Juxta is bony in structure, with *M. teleia* showing greater separation compared to *M. arion*; the base is slender.

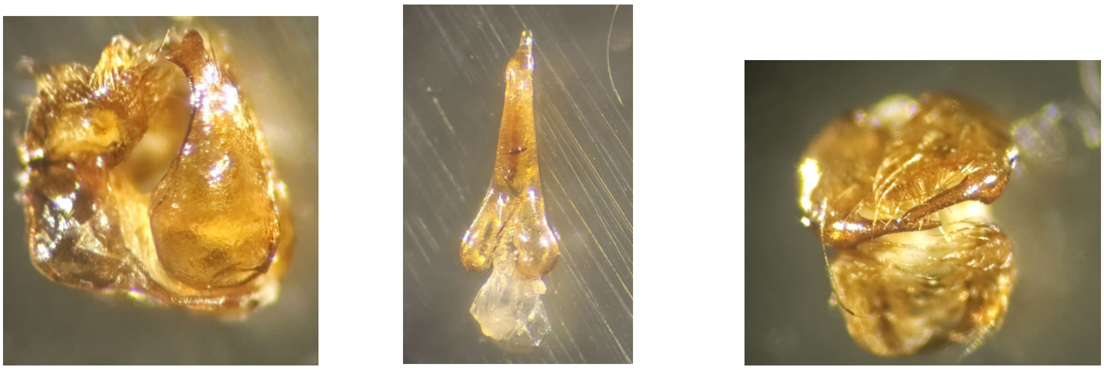

A B C

Figure S2. Male genitalia of *M. arion*

A (lateral view of valva); B (phallus); C (dorsal view of valva);

2. *Maculinea teleia*

This species is small to medium in size, with a dark brown body and a light blue area in the median field. The terminal spots in each median cell are distinctly visible in dark black. The ventral side ranges from light to dark brown, with a series of black spots in the median field clearly observable.

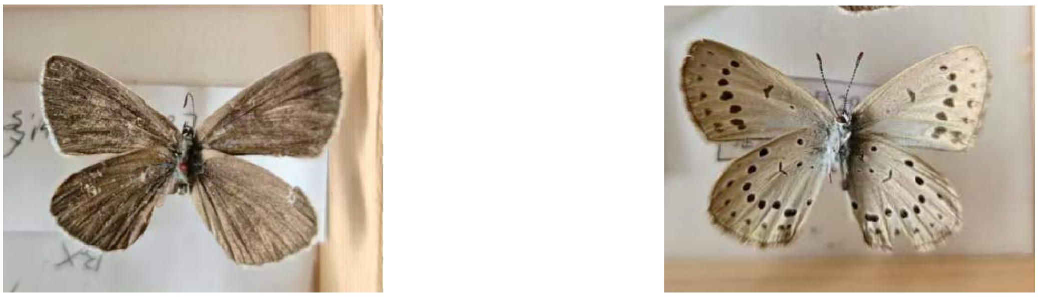

A B

Figure S3. Morphological image of *Maculinea teleia*

A (in frontal view), B (in dorsal view).

The male genitalia feature a large tegumen without a saccus. The valvae are covered with long hairs dorsally, with a shallow depression at the base of the central cleft and a sharply pointed, downward-directed process at the tip. The uncus are similar in morphology. The juxta is bone-shaped with a rounded and blunt base.

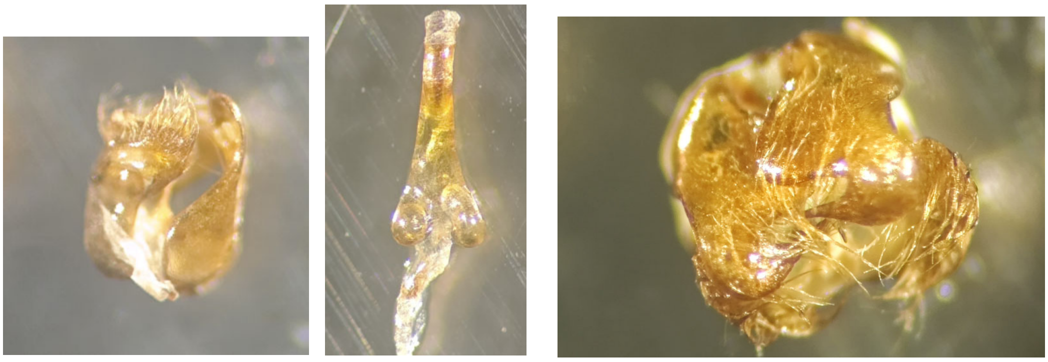

A B C

Figure S4. Male genitalia of *M. teleia*

A (lateral view of valva); B (phallus); C (dorsal view of valva);

Male genitalia comparison of *M. arion* and *M. teleia*.

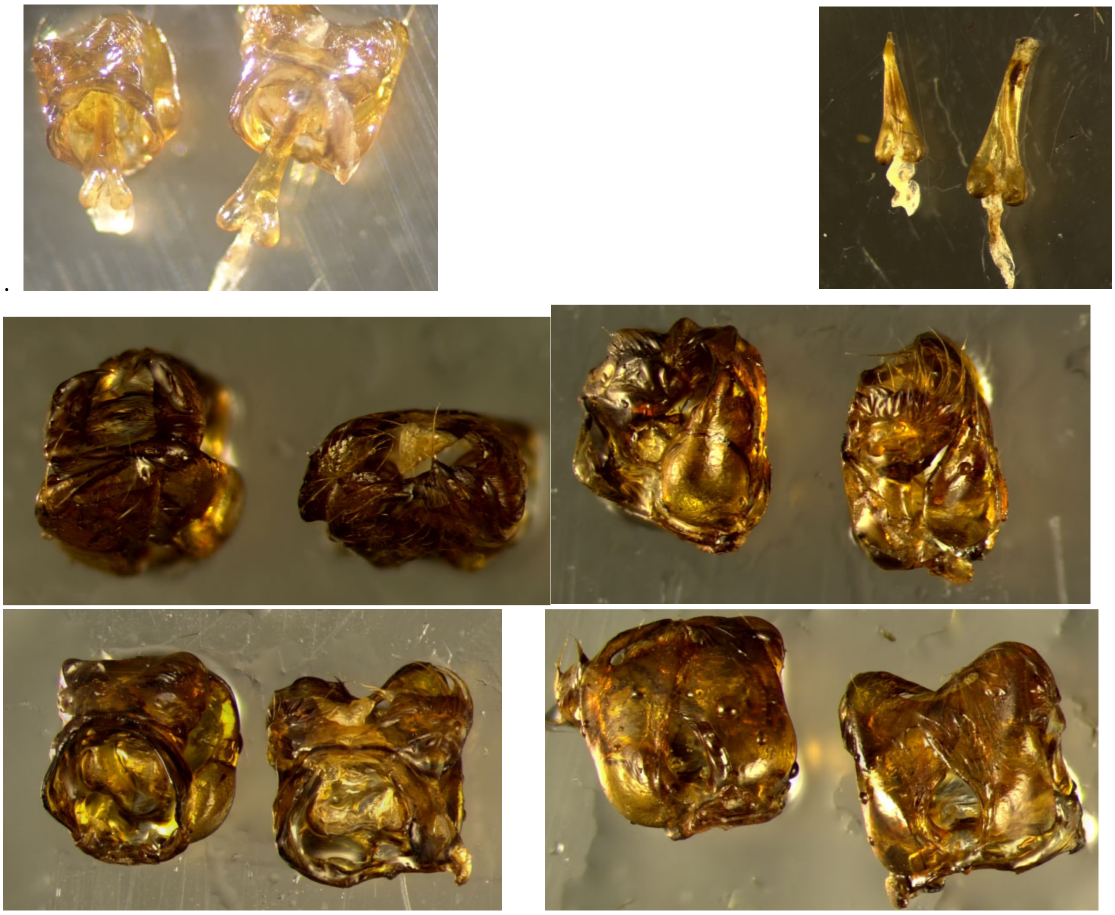

Note: left is ***M. arion*, right is *M. teleia***
